# Hospital Admissions for Personality Disorders Increased During the COVID-19 Pandemic

**DOI:** 10.1177/07067437231155999

**Published:** 2023-02-14

**Authors:** Scott B. Patten, Gina Dimitropoulos, Jeanne V.A. Williams, Sandy Rao, Mina Fahim, Vandad Sharifi, Pardis Pedram, Andrew G.M. Bulloch

**Affiliations:** 1 Department of Community Health Sciences, 2129 University of Calgary, Calgary, Canada; 2 Department of Psychiatry, 2129 University of Calgary, Calgary, Canada; 3 Faculty of Social Work, 2129 University of Calgary, Calgary, Canada; 4 Department of Psychiatry, 48439 Tehran University of Medical Sciences, Tehran, Iran

**Keywords:** borderline personality disorder, eating disorders, mental health services, COVID-19 pandemic

In Canada, the COVID-19 pandemic was associated with more negative self-perceived mental health in the general population, with a greater impact in younger age groups.^1^ A study of Ontarians aged 10+ found a decrease in emergency department visits and hospital admissions soon after the onset of the pandemic, followed by a subsequent return to prepandemic levels.^2^ In distinction, increased hospital admissions for eating disorders have been reported globally and in Canada.^3^

We sought to explore whether increased admissions occurred in diagnostic categories other than eating disorders by obtaining Alberta hospital discharge data for all hospital admissions having a most responsible diagnosis of a mental disorder. We focused on the 10–26 age range (the group in which the pandemic had its greatest impact and where anorexia nervosa (AN) typically has its onset and most severe manifestations) between 1 January 2018 and 31 December 2021. Counting back from the declaration of the global pandemic by the World Health Organization (11 March 2020) we estimated hospital admission rates in 3-month intervals prior to and after 11 March 2020, by dividing the number of hospital admissions within each 3-month interval by the mid-interval provincial population. Admission rates were then stratified by diagnostic group using both ICD-10 chapter categories and by specific diagnostic codes when sample size permitted. The rates were also stratified by age category (10–17 and 18–26) and by sex. We calculated 95% confidence intervals using Wilson's method. We explored linear modelling approaches to describe temporal trends in the data.

Models that regressed hospital admission rates by time had nonsignificant slopes, but plots of standardized residuals for these models consistently identified nonlinear patterns. Whereas some increases were observed, the most recent time intervals (10 June to 9 September 2021 and 10 September to 9 December 2021, corresponding to the 15–21 months postpandemic onset) generally had admission rates resembling prepandemic rates. Due to the nonlinear patterns, a decision was made to describe the temporal patterns graphically rather than through linear modelling or an interrupted time series analysis.

Distinctly increased rates were observed for 2 sets of conditions. First, for eating disorders an increase starting at about 6 months was followed by a gradual reduction in admission rates, trending back towards prepandemic levels. Confidence intervals (95%) for the 6- to 9-month postpandemic interval were distinct from those of prepandemic levels indicating that the difference was unlikely to arise by chance, consistent with prior literature.^3^ Upon stratification, this increase was found to be exclusively due to AN (F50.0) and Atypical AN (F50.1), which is unsurprising since Bulimia Nervosa rarely leads to hospitalization. Stratification by age and sex was unsuccessful due to imprecision in the stratum-specific estimates.

**Figure 1.**
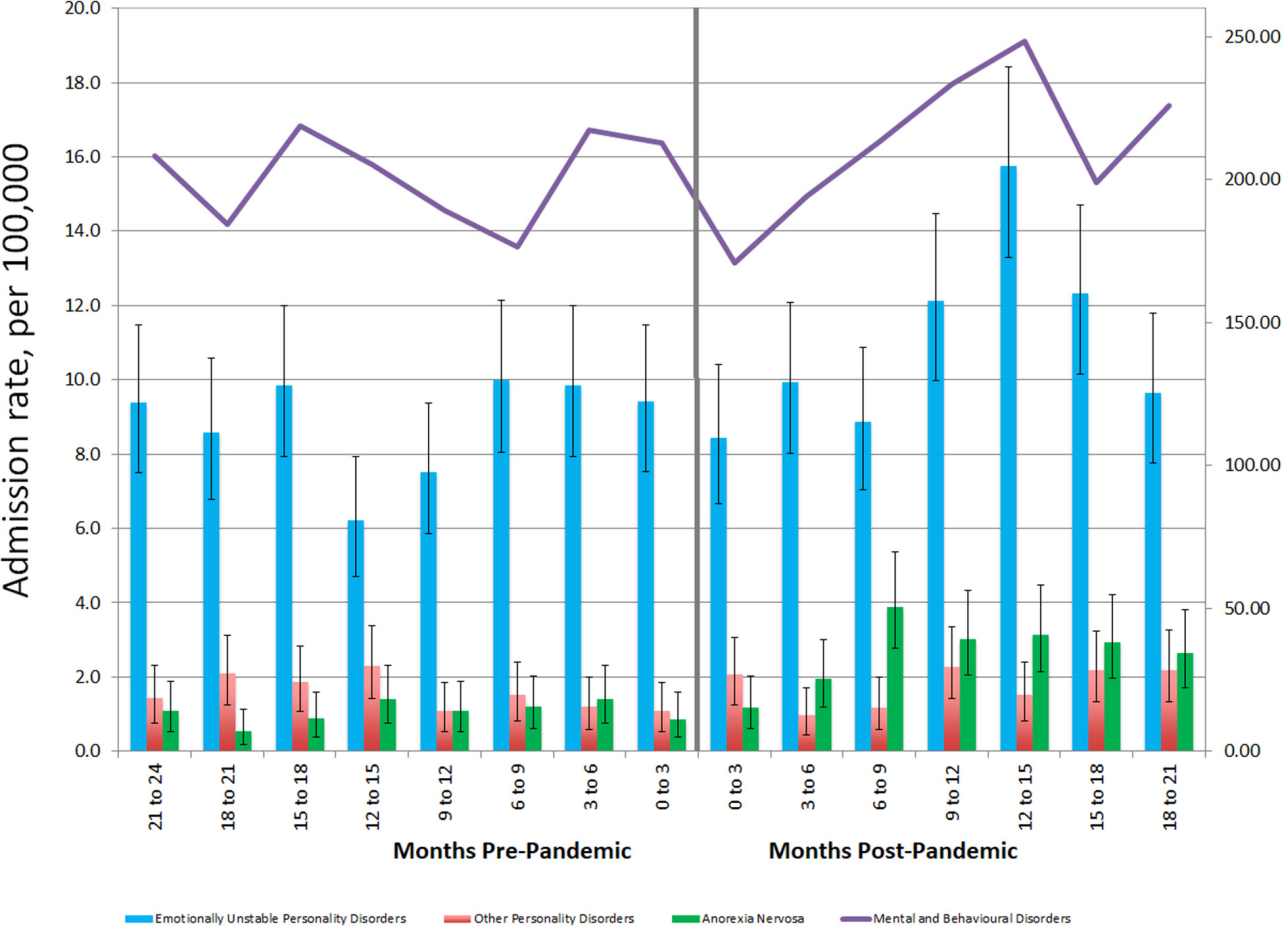
Hospital admission rates for all ICD F-codes (y-axis labels on right, per 100,000), personality disorders and eating disorders (y-axis labels on left), reported in 3-month intervals.

Only one other diagnostic category showed a substantial increase during the pandemic, which was the Personality Disorders category (F60–F69 in ICD-10, Disorders of Adult Personality and Behaviour), which showed a distinct increase after about 9 months. Of admissions in this category, 77% fell within the ICD F60.3 category of Emotionally Unstable Personality Disorders, a code most often used for the DSM-5 category of Borderline Personality Disorder, the category of personality disorder most likely to lead to hospitalization. The pattern of admission over time is depicted in Figure 1. There is no clear reduction in the months following the declaration, but there is a subsequent increase that peaks at 12–15 months postpandemic onset, with confidence intervals distinct from those of prepandemic rates. Again, stratification by age and sex was not informative due to imprecision in the 10–17 age range and in males.

Personality disorders are defined in ICD-10 as “enduring behaviour patterns, manifesting as inflexible responses to a broad range of personal and social situations.” This represents a group whose coping resources may have been limited and who may have been more frequently overwhelmed in a changing and stressful psychosocial context, such as that of a pandemic. Other mechanisms may also contribute such as patterns of substance use or vulnerabilities to rejection or abandonment. Based on the characteristics of these disorders, Preti et al.^4^ hypothesized that the pandemic would be poorly tolerated in people with personality disorders, but a subsequent systematic review found only a few small clinical studies and low-quality surveys.^5^ The analysis presented here suggests that people with personality disorders in Alberta manifested increased rates of hospitalization during early phases of the pandemic. Indeed, the increase was more pronounced than the widely reported increase in admissions for eating disorders. This observation has implications for pandemic preparedness of generic psychiatric inpatient resources, and not merely for the specialized inpatient resources often used by eating disorder patients. For example, in areas where personality disorder admissions are managed in short-stay or crisis support units, these may need increased resources during public health emergencies.

Pardis Pedram is currently affiliated with Faculty of Social Work, University of Calgary, Calgary, Canada
